# AI-Assisted Triage for Diabetic Retinopathy Integrated into Routine Diabetes Care: A Real-World Diagnostic Accuracy Study of the DAIRET^®^ Algorithm

**DOI:** 10.3390/diagnostics16203422

**Published:** 2026-10-21

**Authors:** Alberto Vassallo, Alessio Delfino, Greta Ghizzardi, Andrea Carbone, Gloria Groppelli, Gisella Boselli, Noemi Busca, Matteo Cerutti, Sara Lodigiani, Caterina Golinelli, Guido Grignaffini, Valeria Guazzoni

**Affiliations:** 1 Unit of Diabetology and Endocrinology, ASST di Lodi, Via Largo Donatori del Sangue 60, 26900 Lodi, Italy; andrea.carbone@asst-lodi.it (A.C.); gloria.groppelli@asst-lodi.it (G.G.); gisella.boselli@asst-lodi.it (G.B.); noemi.busca@asst-lodi.it (N.B.); matteo.cerutti@asst-lodi.it (M.C.); sara.lodigiani@asst-lodi.it (S.L.); caterina.golinelli@asst-lodi.it (C.G.); valeria.guazzoni@asst-lodi.it (V.G.); 2 Department of Ophthalmology, ASST di Lodi, Via Largo Donatori del Sangue 60, 26900 Lodi, Italy; alessio.delfino@asst-lodi.it; 3 Healthcare Professions Directorate, ASST di Lodi, Via Fissiraga 15, 26900 Lodi, Italy; greta.ghizzardi@asst-lodi.it; 4 General Directorate, ASST di Lodi, Piazza Ospitale 10, 26900 Lodi, Italy

**Keywords:** diabetic retinopathy, artificial intelligence, screening, diabetes, diagnostic accuracy, triage, STARD-AI

## Abstract

**Background**: Screening for diabetic retinopathy (DR) is resource-intensive. We evaluated the real-world diagnostic accuracy of the DAIRET^®^ Artificial Intelligence (AI) algorithm, integrated into the electronic medical record, as a triage tool in routine diabetes care. **Methods**: Retrospective, STARD-AI-compliant study of consecutive adults with diabetes screened with non-mydriatic fundus photography between April and December 2025. The DAIRET^®^ output (“suspected”/“not suspected”) was compared with grading by an ophthalmologist masked to the AI result. Sensitivity, specificity and predictive values with exact 95% confidence intervals were calculated for any DR and referable (moderate or worse) DR. **Results**: Among 1051 patients, 185 (17.6%) had DR: 160 mild and 25 moderate non-proliferative DR; none had severe DR. DAIRET^®^ classified 271 patients (25.8%) as suspected. For any DR, sensitivity was 59.5% (52.0–66.6) and specificity 81.4% (78.7–83.9). For referable DR, sensitivity was 100.0% (86.3–100.0), specificity 76.0% (73.3–78.6) and negative predictive value 100.0% (99.5–100.0). Specificity was lower in patients aged <65 years and with type 1 diabetes. **Conclusions**: No referable case was missed, although sensitivity was based on 25 cases (lower limit 86.3%) and 15 AI-negative patients lacked complete verification. The 74% classified as “not suspected” is a potential, not measured, reduction in referrals. Performance for severe or proliferative DR could not be assessed, and multi-center confirmation is needed.

## 1. Introduction

Diabetes mellitus (DM) represents one of the fastest-growing global health emergencies of the 21st century, with global prevalence projected to reach over 1.3 billion cases by 2050 [1]. In Italy, population-based data indicate a national prevalence ranging between 4.7% and 6.3%, though integrated analyses often reveal a substantial underdiagnosis rate [2]. Among microvascular complications, diabetic retinopathy (DR) remains the primary cause of preventable blindness among working-age adults [3]. Beyond metabolic pathways, the pathogenesis of both DM and DR is driven by chronic low-grade inflammation. Hyperglycemia sustains oxidative stress, up regulation of pro-inflammatory cytokines and leukostasis, which synergically contribute to the microvascular damage underlying DR [4,5]. Although early-stage DR is asymptomatic, timely detection and management can reduce the 5-year risk of severe vision loss by up to 90% [6]. Consequently, regular screening is universally recognized as a key component of comprehensive diabetes care and an essential public health intervention to prevent individual disability and long-term socioeconomic burden [7].

Despite established clinical guidelines, the implementation of traditional screening pathways suffers from relevant structural and organizational bottlenecks. Conventional screening based on direct fundus examination (DFE) or conventional fundus photography (CFP), reviewed asynchronously by ophthalmologists, is frequently hampered by limited ophthalmological resources and prolonged waiting times, often leading to poor patient adherence and delayed secondary care referrals [8,9]. Furthermore, recent regional healthcare policy shifts in Italy restricting the prescription of standalone DFE outside of a comprehensive ophthalmological consultation compel healthcare providers to decentralize screening delivery directly into diabetology settings. However, screening models that rely exclusively on manual image assessment by the specialists may generate substantial referral volumes, straining ophthalmological resources and worsening institutional backlogs [8].

Artificial Intelligence (AI) technologies integrated into point-of-care workflows have emerged as a promising approach to support the implementation of scalable DR screening. Automated pre-evaluation algorithms can provide an initial assessment, filtering out non-suspected cases at the point of care and ensuring that only suspicious scans are forwarded to ophthalmological evaluation [10]. Globally, various AI algorithms have already demonstrated highly promising results as automated screening tools, achieving robust diagnostic performance across demographic and clinical strata [11,12]. This triage model holds the potential to expand screening coverage, streamline clinical workflows, reduce specialist workloads, and improve health system sustainability [13,14].

Within this framework, the DAIRET^®^ (*Diabetes Artificial Intelligence for RETinopathy*) algorithm offers an automated pre-evaluation tool for retinal images directly integrated into clinical diabetes platforms. The feasibility and diagnostic performance of this approach have previously been explored in an Italian pilot study, providing initial evidence supporting the use of AI-assisted pre-evaluation for DR screening [15]. Nevertheless, the diagnostic performance of AI-based tools may vary according to population characteristics, image acquisition procedures and regional disease prevalence [16]. Therefore, real-world evaluations assessing diagnostic accuracy alongside organizational impacts within routine public health settings remain crucial. This study aims to evaluate the real-world diagnostic performance of the DAIRET^®^ algorithm in a large cohort of patients with DM attending a hospital in Northern Italy, and to explore its potential role as an AI-assisted triage tool within DR screening pathways integrated into routine diabetes care.

## 2. Materials and Methods

### 2.1. Study Design and Participants

We conducted a retrospective diagnostic-accuracy study at the Diabetology Unit of ASST Lodi (Lombardy, Italy), reported in accordance with the STARD-AI guideline [17] (checklist in Supplementary Materials Table S1). All consecutive adults (≥18 years) with diabetes mellitus (DM) of any type who underwent CFP for DR screening between 8 April and 10 December 2025 were eligible. The primary exclusion criterion was a pre-existing clinical diagnosis of DR, as these patients were already enrolled in secondary specialist care, and therefore did not enter the primary screening pathway. No pre-selection was performed regarding other ocular comorbidities (such as macular disorders, refractive abnormalities or hypertensive retinal changes), reflecting an unselected real-world screening cohort. Examinations lacking the index test output or a complete ophthalmologist’s report were excluded from the accuracy analysis (Figure 1). The unit of analysis was the patient: each patient contributed a single examination, classified according to the more severely affected eye. No formal sample size calculation was performed; all eligible examinations in the study period were included, and the precision achieved is reported as 95% confidence intervals. Retinal images and clinical data analyzed in this study were originally generated during routine clinical practice between April and December 2025 as a part of a regional healthcare planning program. When secondary use of these clinical data for research purposes was subsequently planned, the protocol was submitted to the Territorial Ethics Committee Lombardia 1, which issued a favorable opinion on 22 April 2026. No analysis was performed before the formal approval was obtained. Written informed consent for participation to the study and use of previously collected clinical data was obtained from all patients included in the analysis at their first scheduled visit following ethical approval.

### 2.2. Image Acquisition and Data Collection

Minimal demographic data (age, sex, type of diabetes and disease duration) were extracted from the electronic medical record (EMR). Fundus photograph acquisition was integrated into routine diabetology visits. Retinal images were acquired using a non-mydriatic desktop digital fundus camera (DRSplus^®^, CenterVue S.p.A., Padova, Italy) located adjacent to the outpatient diabetology clinics. Fundus photographs were acquired by trained healthcare professionals without pharmacological pupil dilation as part of routine clinical visits, according to a standard protocol of two fields per eye (45°, macula-centered and optic disc-centered). Following the acquisition, the retinal photographs were stored directly within the MètaClinic^®^ electronic medical record (EMR) platform. No formal image-quality grading step was applied before analysis: images were not pre-screened, rejected or re-acquired on the basis of quality, and all acquisitions obtained during the study period were submitted both to the algorithm and to the human grader. Neither the algorithm nor the grader used an “ungradable” category, so image quality is reflected in the resulting classifications rather than in a separate exclusion step (Section 2.3 and Section 3.1).

### 2.3. Index Test: DAIRET^®^ Algorithm

Retinal image pre-evaluation was performed using DAIRET^®^ (Meteda S.r.l., San Benedetto del Tronto, Italy) version 2.0, an automated machine-learning software nested directly within the EMR platform. DAIRET^®^ represents the commercial Italian implementation of Retmarker Screening (Retmarker SA, Coimbra, Portugal), a CE-marked Class IIa medical device [18]. The underlying algorithm combines classical image-processing steps with deep-learning-based lesion detection [19] and was trained in 2015 and 2018 on a large multi-center dataset comprising over 55,000 patients and 245,000 retinal images [15,20].

Following imaging acquisition, non-mydriatic fundus photographs uploaded to the EMR network were processed by the algorithm to detect elementary lesions of DR (including microaneurysms, retinal hemorrhages, and exudates). The software operates on a fixed binary decision logic with a manufacturer-set operating threshold that cannot be modified by the user: if features suggestive of DR are detected, the system automatically generates an alert message classifying the scan as “suspected”; otherwise, it is flagged as “not suspected”. The software returns a single output per examination (patient level), which was the unit recorded in this study. The manufacturer does not document how the four images acquired per patient (two fields per eye) are combined into this single output, and the aggregation rule is neither reported to nor configurable by the user; we recorded the output as issued by the software and, in accordance with STARD-AI, report this lack of documentation explicitly. The patient-level output is nonetheless aligned with the reference standard, which was also recorded per patient according to the more severely affected eye. It does not return a continuous probability score to the user; consequently, only a single operating point could be evaluated, and a receiver operating characteristic (ROC) curve could not be constructed. The AI pre-evaluation relied exclusively on the retinal images, without access to clinical information or to the ophthalmologist’s report. In the intended use evaluated here, images are acquired by nurses or other healthcare professionals without ophthalmological training, and the output is read by the diabetologist during the visit, who refers “suspected” patients for ophthalmological assessment. Further details on network architecture, the composition of the training and tuning datasets and image preprocessing are proprietary and have not been disclosed by the manufacturer; in accordance with STARD-AI [17], we report this explicitly. The software was used in its fixed, certified commercial configuration (version 2.0) throughout the study period, without local retraining, recalibration or threshold adjustment.

### 2.4. Reference Standard

Parallelly, all retinal images were graded asynchronously by an ophthalmologist (human grader defined as the Reference Standard) from the Eye Clinic of ASST Lodi, who was blinded to the AI outputs. The grader, a consultant ophthalmologist with over fifteen years of experience in clinical ophthalmology, graded the images on the same EMR platform, masked to the AI output, within fourteen days of acquisition; since the index test and the reference standard were applied to the same images, no clinical intervention could occur between the two assessments. Ophthalmological assessment was considered the reference standard. The human grader classified the presence and severity of DR according to the International Clinical Diabetic Retinopathy (ICDR) Severity Scale (no apparent retinopathy, mild, moderate or severe non-proliferative DR (NPDR), or proliferative DR (PDR) [21]. Moderate or worse DR was defined a priori as “Referable” DR (i.e., requiring specialist ophthalmological assessment). Diabetic macular edema (DME) and diabetic maculopathy were not graded separately: the reference standard applied the ICDR severity scale only, and the two-field non-mydriatic color photography used in this pathway, without optical coherence tomography or stereoscopic imaging, do not allow their reliable detection. “Referable” DR in this study therefore denotes moderate or worse DR and does not encompass referable macular disease. Grading was recorded per patient, according to the more severely affected eye. Ophthalmological grading is the reference standard adopted in routine screening at our institution; a single-grader reference standard has also been adopted in previous real-world evaluations of AI-based DR screening, including the pilot study of the same software [15,22]. Finally, the diagnostic performance of the AI algorithm was evaluated by comparing its binary output directly with the ophthalmologist’s clinical report. A participant flow diagram drawn according to STARD-AI is shown in Figure 1.

### 2.5. Statistical Analysis

Descriptive statistics were used to summarize the baseline characteristics of the study cohort. Continuous variables were tested for normality using the Shapiro–Wilk test. Normally distributed continuous variables were expressed as mean ± standard deviation (SD), whereas non-normally distributed variables were reported as median and interquartile range (IQR). Categorical variables were presented as absolute frequencies and percentages (n, %). The diagnostic performance of DAIRET^®^ was evaluated against the reference standard by calculating sensitivity, specificity, positive predictive value (PPV), negative predictive value (NPV), and overall accuracy, with 95% confidence intervals (CIs) estimated using the exact Clopper–Pearson method for every estimate, including subgroup estimates. Analyses were conducted for both any DR and referable DR (moderate or worse). The proportion of patients classified as “not suspected” was reported as an estimate of the potential reduction in ophthalmological referrals. When no events were observed (e.g., severe DR), the upper limit of the exact 95% CI is reported. Agreement between the AI algorithm and the ophthalmologist was assessed using unweighted Cohen’s kappa coefficient (κ) with asymptotic 95% CI, interpreted according to Landis and Koch [23]. Exploratory subgroup analyses were performed by age (<65 vs. ≥65 years), sex, type of diabetes (type 1, type 2, other) and diabetes duration (<10 vs. ≥10 years). Difference in specificity between subgroups were tested using Fisher’s exact test. To account for potential confounding by age when comparing diabetes types, an age-stratified Mantel–Haenszel analysis was conducted. Patients with missing covariates were retained in the main analysis and excluded only from the corresponding subgroup (age, n = 3; sex, n = 1; duration, n = 2). As an error analysis, the causes of false-positive classifications documented in the ophthalmologist’s report were extracted and categorized. A sensitivity analysis addressed partial verification, by recalculating diagnostic metrics under the assumption that 1, 2, 3 or all 15 AI-negative patients without a complete reference standard had referable DR, and across all 33 patients lacking a complete reference standard. No indeterminate index-test results occurred. All statistical analyses were conducted using Python 3.11 (pandas 3.0 and SciPy 1.17).

## 3. Results

Between 8 April and 10 December 2025, 1085 examinations were performed; 33 lacked a complete ophthalmologist’s report and one lacked the AI output, leaving 1051 patients for the accuracy analysis (Figure 1). Of these, 668 (63.6%) were males (sex missing in 1). The median age was 64.6 years (IQR 56.3–72.8; mean 63.8 ± 13.3), and 514 patients (49.0%) were aged ≥65 years. The majority of patients (91.4%) had type 2 diabetes (T2D), followed by type 1 diabetes (T1D) (7.3%) and other specific types (1.2%: pancreatogenic, n = 6; steroid-induced, n = 7). The median duration of diabetes was 7 years (IQR 2–14) (Table 1).

### 3.1. Prevalence of Diabetic Retinopathy (DR)

The algorithm does not provide an “ungradable” output category: every examination returned either a “suspected” or a “not suspected” classification, and no examination was rejected for insufficient image quality by the human grader either. Images of reduced quality were therefore classified rather than excluded, and were predominantly flagged as “suspected”. According to the ophthalmologist assessment, DR was absent in 866 patients (82.4%). DR of any grade was diagnosed in 185 patients (17.6%; 95% CI 15.3–20.0): 160 (15.2%) presented with mild NPDR and 25 (2.4%) with moderate NPDR. No cases of severe NPDR or PDR were observed (0/1051; upper limit of the 95% CI 0.35%). Conversely, the AI algorithm classified 25.8% of the acquisitions (n = 271) as “suspected” for DR, designating the remaining 74.2% (n = 780) as “not suspected” (Table 2).

### 3.2. Overall Diagnostic Performance for DR (Of Any Severity)

Compared with the ophthalmologist’s report for detecting any grade of DR, the AI algorithm showed an overall sensitivity of 59.5% (110/185; 95% CI, 52.0–66.6). Overall specificity was 81.4% (705/866; 95% CI 78.7–83.9). The positive predictive value (PPV) was 40.6% (110/271; 95% CI 34.7–46.7), whereas the negative predictive value (NPV) reached 90.4% (705/780; 95% CI 88.1–92.4). The overall accuracy of the algorithm was 77.5% (815/1051; 95% CI 74.9–80.0). The degree of concordance between the AI output and the clinical evaluation indicated a fair agreement (κ = 0.35; 95% CI 0.27–0.42) (Table 3).

The operating points for any DR and referable DR are shown in ROC space in Figure 2; because the software provides only a binary output, a full ROC curve and an area under the curve could not be derived. Representative non-mydriatic fundus images depicting the diagnostic classification outcomes of the AI algorithm—including True Negative, True Positive, False Negative, and False Positive cases—are illustrated in Figure 3.

**Figure 2:**
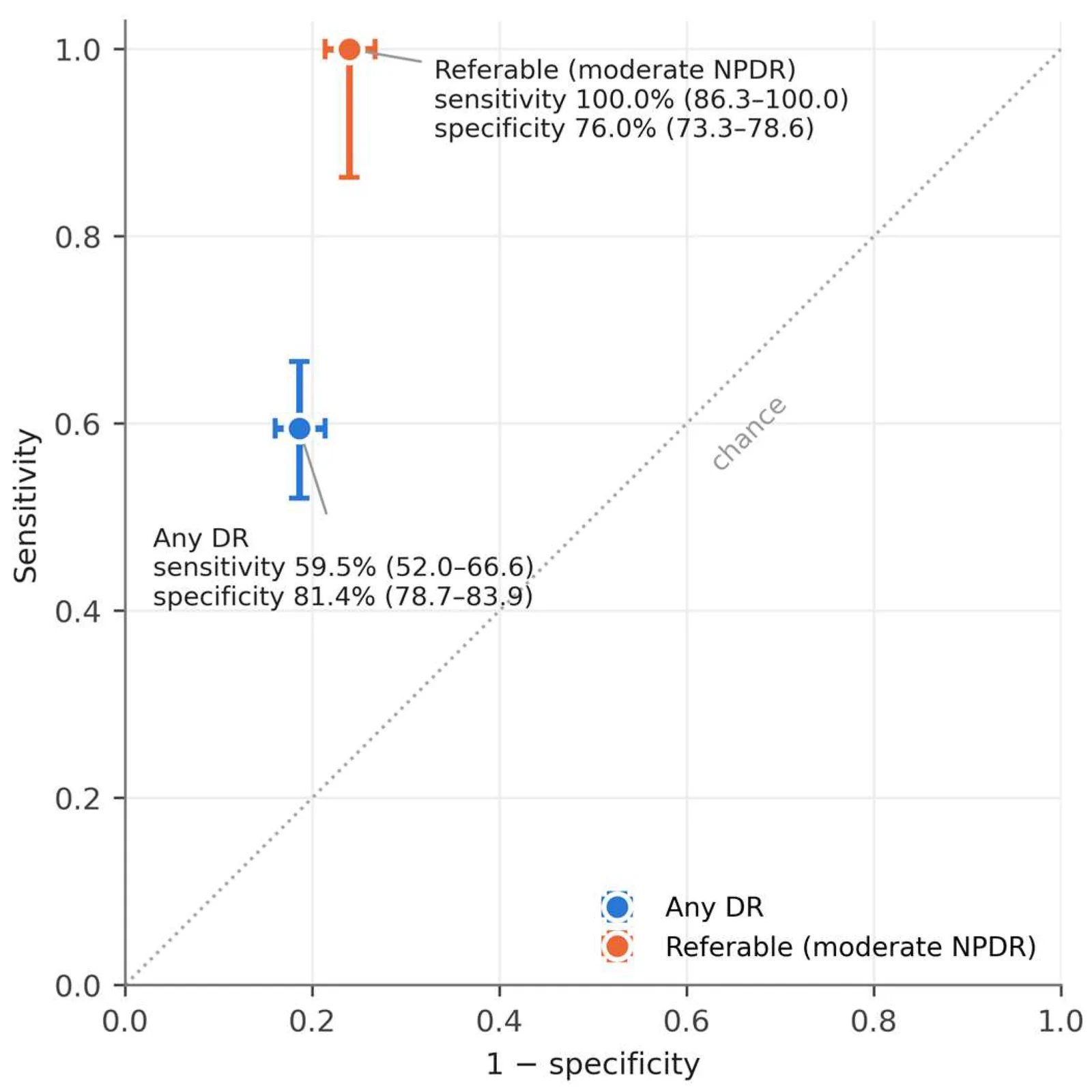
Operating points of DAIRET^®^ in receiver operating characteristic (ROC) space for any DR (blue) and referable DR (orange). Error bars show exact 95% CIs for sensitivity (vertical) and 1–specificity (horizontal). Because the software returns only a binary output, a single operating point per target condition is available and no ROC curve or area under the curve can be derived.

**Figure 3:**
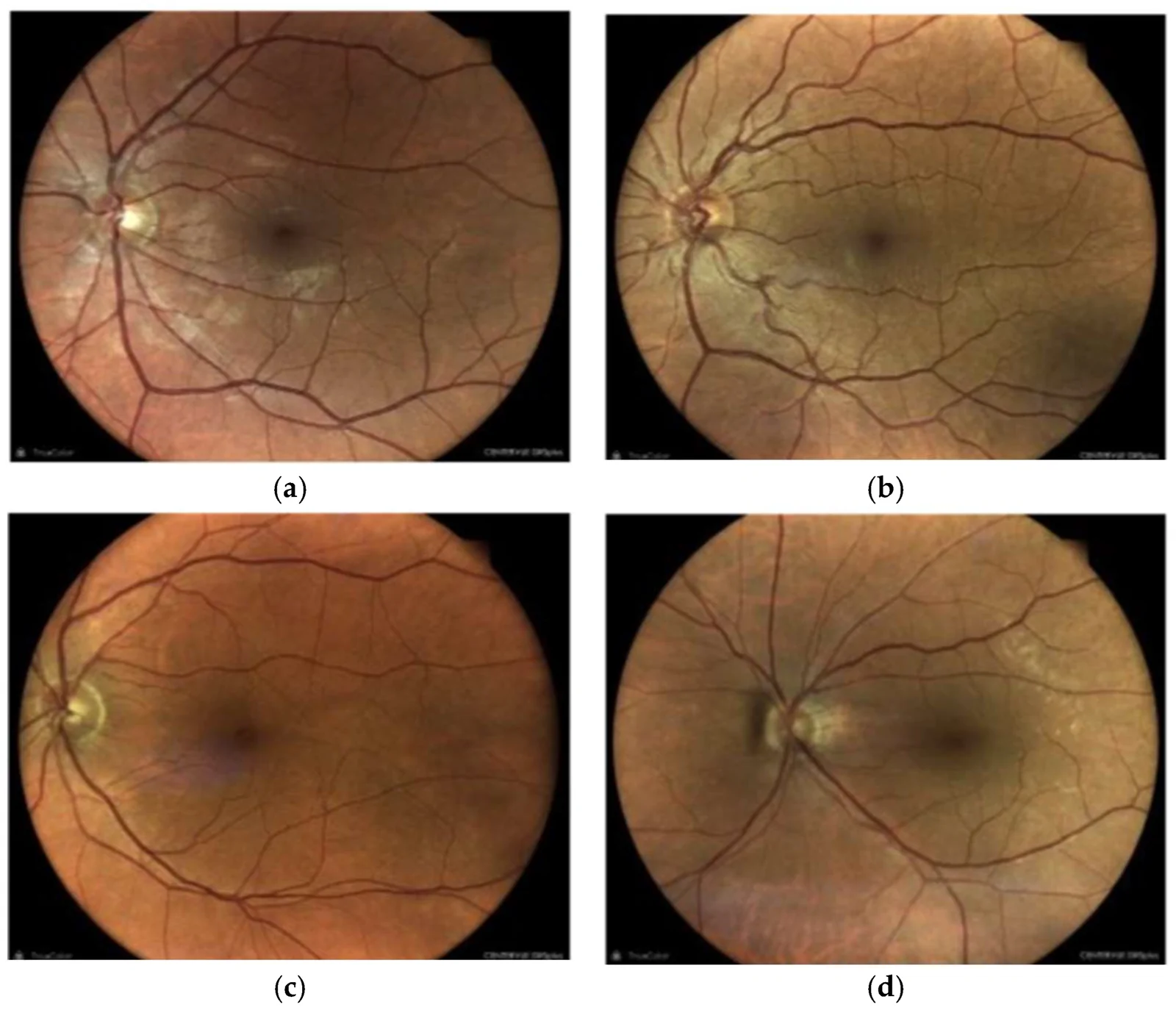
Representative non-mydriatic fundus photographs (left eyes) from the study cohort illustrating the diagnostic classification of the DAIRET^®^ AI algorithm compared to the ophthalmological reference standard: (**a**) True Negative: Classified as “not suspected” by AI and confirmed as “Absent DR” by the ophthalmologist, displaying normal retinal vascular architecture, a clear macula, and no microvascular abnormalities. (**b**) True Positive: Classified as “suspected” by AI and confirmed as DR by the ophthalmologist, demonstrating elementary microvascular lesions, including isolated microaneurysms and subtle dot hemorrhages along the vascular arcades. (**c**) False Negative: Classified as “not suspected” by AI but graded as mild NPDR by the ophthalmologist, characterized by a solitary, low-contrast microaneurysm against a tessellated fundus background (all false negatives in the study were strictly confined to mild, non-referable DR cases). (**d**) False Positive: Classified as “suspected” by AI but graded as “Absent DR” by the ophthalmologist, showing high-contrast vitreoretinal interface reflections (cellophane sheen) temporal to the macula, which were the most likely trigger of the alert (the software does not indicate which image feature determined the output).

### 3.3. Diagnostic Performance for Referable DR

The severity-stratified analysis revealed distinct performance levels depending on the degree of DR. For referable DR, the AI achieved a sensitivity of 100.0% (25/25; 95% CI 86.3–100.0), recognizing all 25 cases of moderate DR present in the sample. No severe DR cases were observed, and therefore the performance of the algorithm for severe NPDR or PDR could not be estimated. Specificity in differentiating moderate cases from those with absent or mild DR was 76.0% (780/1026; 95% CI 73.3–78.6). False negatives for any DR (n = 75) were entirely attributable to mild forms, with the algorithm failing to flag 75 of 160 cases of mild DR (46.9%), while no case of referable DR (0/25) was labelled as not suspicious by the algorithm. Finally, 161 false positives were recorded among patients without DR, corresponding to 18.6% of patients evaluated as free of DR by the ophthalmologist (Table 2 and Table 3).

For referable DR the PPV was 9.2% (25/271; 95% CI 6.1–13.3), reflecting the algorithm’s high sensitivity threshold which also captures mild cases and false positives. Conversely, the NPV for referable DR reached 100.0% (780/780; 95% CI 99.5–100.0), indicating that, in this cohort, a “not suspected” output was never associated with referable disease. Overall accuracy for the detection of referable DR was 76.6% (805/1051; 95% CI 73.9–79.1). Agreement between AI output and the ophthalmological reference assessment was slight (κ = 0.13; 95% CI 0.04–0.23), reflecting the low prevalence of the target condition in the study population (2.4%) (Table 3, Figure 2).

### 3.4. Subgroup Analyses

Across subgroups, no patient with referable DR was classified as “not suspected” (sensitivity and NPV for referable DR 100% in every stratum), although the lower confidence limits were wide where referable cases were few (e.g., women, five cases, lower limit 47.8%). Specificity for referable DR was lower in patients aged <65 years than in those aged ≥65 years (70.0% vs. 82.2%; Fisher’s exact test *p* < 0.001) and in T1D compared with T2D (50.0% vs. 78.0%; Fisher’s exact test *p* < 0.001). Because T1D patients were considerably younger (median age 43.9 vs. 65.7 years; 85.7% vs. 48.0% aged <65 years), the comparison was repeated with stratification by age: the Mantel–Haenszel odds ratio (OR) of an AI-positive result in T1D versus T2D was 2.87 (95% CI 1.74–4.73, *p* < 0.001), indicating that the difference was not explained by age alone. However, the association was driven by the <65 years stratum (specificity 44.4% vs. 73.3%, *p* < 0.001), with no appreciable differences among those aged 65 years or over (specificity 90% vs. 82.1%, *p* = 1.00). These subgroup comparisons were not pre-specified and are reported as exploratory. As a consequence, the proportion of AI-positive results was higher in younger patients (32.4% vs. 18.9%) and in T1D (51.9% vs. 23.8%). Sex and diabetes duration were not associated with appreciable differences in specificity for referable DR, although both were associated with specificity for any DR (*p* = 0.011 and *p* = 0.010, respectively) (Table 4, Figure 4).

### 3.5. Error Analysis of False-Positive Classifications

Among the 161 patients without DR classified as “suspected”, the ophthalmologist documented an alternative finding in 80 (49.7%) (Table 5). Image artefacts (n = 22) and physiological light reflexes (n = 17) were the most frequent single causes; reflex-related false positives occurred predominantly in young patients (median age 33 years). Non-DR retinal or optic-disc findings that may themselves warrant ophthalmological assessment were reported in 35 patients (21.7%), mainly epiretinal membranes (n = 13), drusen or other retinal pigment epithelium changes (n = 8), vascular changes including two sequelae of branch retinal vein occlusion (n = 5), and myopic or atrophic changes (n = 5). For 81 false positives (50.3%), no alternative finding was reported.

**Table 5:** Causes of false-positive AI classifications among patients without DR (n = 161), as documented in the ophthalmologist’s report.

Cause	n	% of False Positives
Image artefacts	22	13.7
Physiological light reflexes (retinal, foveal, corneal)	17	10.6
Epiretinal membrane/macular pucker/cellophane maculopathy	13	8.1
Drusen, RPE changes, macular degeneration	8	5.0
Myopic fundus, peripapillary or chorioretinal atrophy	5	3.1
Vascular changes (hypertensive retinopathy, vessel tortuosity, BRVO sequelae) ^a^	5	3.1
Media opacities (vitreous, corneal, lens)	4	2.5
Isolated retinal hemorrhage not graded as DR	2	1.2
Myelinated nerve fibers	2	1.2
Optic disc cupping	1	0.6
Chorioretinal scar	1	0.6
Not specified in the report	81	50.3
Total	161	100

RPE = retinal pigment epithelium; BRVO = branch retinal vein occlusion. ^a^ Two BRVO sequelae, two vessel tortuosity, one hypertensive retinopathy. One patient with macular pucker and a suspected macroaneurysm is counted under epiretinal membrane. Classification was based on free-text annotations and assigns each patient to one category.

### 3.6. Sensitivity Analyses

AI-positive results were over-represented among the 33 patients without a complete reference standard (18/33, 54.5%, vs. 25.8% in the analyzed cohort). If one, two or three of the 15 AI-negative patients without a complete reference standard had referable DR, sensitivity for referable DR would be 96.2% (95% CI 80.4–99.9), 92.6% (75.7–99.1) and 89.3% (71.8–97.7), respectively, with NPV ≥ 99.6% (Supplementary Materials Table S2). Varying the reference standard across all 33 unverified patients, rather than the 15 AI-negative ones only, also affects specificity and positive predictive value: across the extreme scenarios specificity ranged from 74.7% to 76.4% and PPV from 8.7% to 14.9%, while sensitivity ranged from 62.5% in the worst case to 100% (Supplementary Materials Table S3).

## 4. Discussion

In this diagnostic accuracy study, we assessed the real-world clinical performance of an AI-based pre-evaluation software (DAIRET^®^) integrated directly into routine diabetology workflows for the triage of DR. Among 1051 consecutive diabetic patients, no case of referable DR was missed, which in this cohort consisted exclusively of moderate NPDR (sensitivity 100%, 95% CI 86.3–100; NPV 100%, 95% CI 99.5–100). This estimate carries appreciable uncertainty, which should be considered in its interpretation: with only 25 referable cases the lower confidence limit of sensitivity is 86.3%, and 15 AI-negative patients lacked a complete reference standard, so that a single missed referable case among them would reduce sensitivity to 96.2% (Supplementary Materials Table S2). About three-quarters of patients (74.2%) were classified as “not suspected”, and none of those with a complete reference standard had referable disease. Within these limits, these findings support the potential role of the algorithm as a high-sensitivity triage tool for referable disease within an integrated diabetes–ophthalmology pathway, rather than as a comprehensive screening test for DR of any severity.

By embedding the DAIRET^®^ algorithm directly into the EMR software used during routine diabetology visits, our study supports a practical model of integrated retinal screening to the point of care, where AI may promptly support the identification of patients requiring further specialist assessment. Non-ophthalmologic healthcare personnel can acquire fundus photographs at the point of care, and the automated system immediately filters out clear scans. In our cohort of 1051 patients, the algorithm flagged 271 cases (25.8%) as “suspected” and cleared 780 cases (74.2%) as “not suspected”. By filtering a substantial proportion of patients who are unlikely to have clinically significant DR, this approach may allow specialist ophthalmologists to prioritize the evaluation of patients with suspected retinal abnormalities [24,25]. From a public health perspective, this model may be particularly relevant in healthcare systems in which the increasing prevalence of diabetes is accompanied by limited ophthalmological capacity [26]. This aligns with public health strategies aimed at enhancing comprehensive diabetes care and early prevention for chronic complications [7,24]. Systematic reviews and meta-analyses have highlighted the potential of AI-assisted DR triage to improve screening accessibility, efficiency, and resource allocation [11,12,27,28], while also showing that AI-supported screening pathways may increase patient adherence and referral follow-up uptake by reducing reporting delays and enabling immediate clinical counselling during routine visits [29,30]. Although our study did not directly measure operational outcomes or healthcare costs, our findings are consistent with this broader evidence and further suggest that integrating AI-assisted pre-evaluation into routine diabetic care may represent a practical approach to support more efficient allocation of ophthalmological resources [13,14,31].

When contextualized within the existing literature, the diagnostic performance of DAIRET^®^ should be interpreted in light of its distinct, conservative operating profile. Landmark validation studies of algorithms such as IDx-DR (sensitivity 91.0%, specificity 84.0%), EYEART (sensitivity 91.7%, specificity 91.5%), and RETMARKER (sensitivity 85.0%) have reported balanced performance metrics in controlled or mass screening settings [18,20,32]. In contrast, in our real-world cohort, DAIRET^®^ demonstrated a lower sensitivity for any DR (59.5%; 95% CI 52.0–66.6), though consistent with recent real-world evaluations of the same software in Italian diabetology settings (~69%) [15]. Variation in performance across these studies is to be expected owing to differences in graders, disease distribution, and reference standards. This limited sensitivity for early stages must be interpreted with care: the present study assessed diagnostic accuracy only and did not measure patient safety or clinical outcomes, which were beyond its scope. In mild DR the primary therapeutic goal is optimizing metabolic and blood pressure control [6,7]. Because mild DR is asymptomatic and not immediately vision-threatening, these patients remain within the routine annual screening program, in order for progression to be intercepted at a subsequent round. Mild DR is nevertheless not clinically irrelevant: it is an established marker of microvascular disease and may influence the recommended surveillance interval, so that failure to detect it entails a loss of prognostic information even where no immediate ophthalmological intervention is indicated. Across screening programs and guidelines the principal safety requirement for a first-line test is the reliable detection of referable DR, although no single sensitivity threshold is universally established. DAIRET^®^ identified all 25 cases of referable DR (sensitivity 100%); however, the lower confidence limit (86.3%) indicates that, with the number of referable cases available, a true sensitivity similar to that reported for other AI systems cannot be excluded, and the point estimate should not be interpreted as evidence of superiority [12,33]. These findings suggest that the algorithm may be better positioned as a triage tool aimed at identifying patients unlikely to have clinically significant disease rather than as a stand-alone diagnostic system for all stages of DR. In a first-line triage framework, prioritizing maximum sensitivity for diseases requiring prompt specialist management (referable DR) over mild lesions that do not require urgent intervention is a defensible public health strategy, provided that patients classified as “not suspected” remain within a scheduled re-screening program [10,11]. The lower specificity (76.0%) and positive predictive value (9.2%) for referable DR must be interpreted within the context of our unselected real-world study design. In contrast to controlled validation studies that often apply eligibility criteria or pre-evaluate image quality for non-DR ocular pathologies, our cohort comprised all consecutive unselected diabetic patients attending routine screening, without excluding co-existing macular conditions (e.g., age-related macular degeneration, drusen, or epiretinal membranes), refractive defects (e.g., myopic changes) or hypertensive vascular signs. In an automated binary classification system, non-DR retinal alterations may pose diagnostic pitfalls, leading to a “suspected” AI output and thereby lowering the DR-specific PPV. While these cases are formally classified as false positives for DR in diagnostic accuracy analyses, from a screening perspective, this should not necessarily be interpreted as evidence of inappropriate referral, as the identification of an abnormal retinal image that may warrant specialist evaluation could represent a potentially beneficial outcome rather than a screening failure [34,35]. Indeed, in our cohort, 35 of the 161 false positives in patients without DR (21.7%) had non-DR retinal or optic-disc findings documented by the ophthalmologist—mainly epiretinal membranes, drusen or other pigment-epithelium changes, vascular changes including two sequelae of branch retinal vein occlusion, and one optic disc cupping—which may themselves warrant ophthalmological assessment (Table 5). Conversely, image artefacts and physiological light reflexes accounted for about one quarter of false positives; the latter occurred mainly in young patients, plausibly explaining the lower specificity observed in patients aged <65 years and with T1D (Table 4, Figure 4), consistent with previous findings in the literature reporting higher rates of false positive in younger patients [36]. For half of the false positives no alternative finding was reported, which limits this error analysis. Additionally, the low PPV is inherently influenced by the low baseline prevalence of referable DR in our primary screening population (2.4%), as predictive values are highly sensitive to disease pre-test probability. Similarly, the statistical interpretation of inter-rater agreement for the referable DR subset requires epidemiological contextualization. The low Cohen’s κ observed for referable DR (κ 0.13) is only partly explained by the low prevalence of the condition (2.4%), which is known to depress κ even when sensitivity is high (the “kappa paradox”) [37]. The marginal distribution of the classifications also contributes: the algorithm labelled 25.8% of patients as “suspected” against a referable prevalence of 2.4%, so that the large number of false-positive classifications (n = 246) lowers agreement beyond the effect of prevalence alone. Under such epidemiological conditions, sensitivity, specificity, and negative predictive value (100%) offer a far more robust and clinically meaningful assessment of first-line screening performance. For DR of any severity, agreement was fair (κ 0.35; 95% CI 0.27–0.42), consistent with the limited sensitivity of the algorithm for mild lesions.

From an implementation perspective, the subgroup analysis indicates that the observed sensitivity and NPV for referable DR remained 100% across age, sex, diabetes type and duration strata, although the wide confidence intervals resulting from small numbers of referable cases in some subgroups (such as women and T1D) preclude definitive conclusions regarding uniform safety. Conversely, the referral workload varied substantially: in patients younger than 65 years and in those with T1D, one in three and one in two patients, respectively, were flagged as “suspected”. This pattern, together with the frequency of reflex-related false positives in young patients, suggests that local protocols might consider targeted image review before referral in these groups; such strategies should be evaluated prospectively.

Several limitations should be acknowledged. While representative of regional public health settings, the single-center nature of the study may limit the generalizability of the findings to other healthcare systems and patient populations. First, the reference standard relied on a single ophthalmologist, which prevented the evaluation of inter-observer variability among human graders. This approach, which reflects routine screening practice at our institution, has also been adopted in previous real-world evaluations of AI-based DR screening, including the pilot study of the same software [15] and a primary-care evaluation [22]. Although the grader was experienced, masked to the AI output and used a standardized international scale, misclassification in the reference standard—particularly between mild and moderate DR, where inter-grader agreement is lowest—may have biased accuracy estimates in either direction. Second, no severe NPDR or PDR was observed (0/1051; upper 95% confidence limit 0.35%), a structural consequence of excluding patients with known DR from the screening pathway; our findings therefore apply to the detection of moderate NPDR and cannot be extrapolated to sight-threatening disease. Third, the referable cohort was small (n = 25), so that the lower 95% confidence limit of sensitivity was 86.3%. Fourth, 33 patients (3.0%) had no complete ophthalmologist report (21 with no report available and 12 in whom DR was documented without a specified grade) and AI-positive results were over-represented among them (18/33); two of the 15 AI-negative patients in this group were patients in whom DR had been documented but not graded, so that the probability of referable disease among them is higher than the cohort prevalence; if even one of the 15 unverified AI-negative patients had referable DR, sensitivity would fall to 96.2% (Supplementary Materials Table S2). Fifth, the algorithm provides only a binary output, precluding ROC analysis and the evaluation of alternative operating thresholds, and several technical details of the software are proprietary, including the rule by which the images acquired per patient are combined into a single patient-level output. Sixth, diabetic macular disease was not assessed: it was not graded by the reference standard, and the two-field non-mydriatic color photography used in this pathway cannot reliably detect it in the absence of optical coherence tomography, so the present results do not cover referable macular disease. Finally, the actual impact on organizational aspects or formal health economic modeling was not assessed, as falling beyond the primary scope of this accuracy study.

Despite these limitations, the primary strength of this study lies in its evaluation of an AI algorithm integrated into routine clinical software within an unselected, consecutive cohort of diabetic patients in a real-world public hospital setting. This design limits selection bias and reflects the performance to be expected in this particular routine diabetology screening population. It does not, however, confer resistance to spectrum bias: the absence of severe NPDR and PDR is itself a substantial spectrum limitation, and the estimates reported here should not be extrapolated to populations in which sight-threatening disease is represented. If implemented within routine diabetes care, AI-assisted DR triage could streamline patient pathways, improve continuity between diabetology and ophthalmology services, and contribute to a more efficient allocation of human and economic resources.

## 5. Conclusions

Integration of the DAIRET^®^ algorithm into routine diabetology workflows offers a feasible and promising model for diabetic retinopathy triage. In this real-world cohort, a “not suspected” output was never associated with referable DR among patients with a complete reference standard, although the lower confidence limit of sensitivity (86.3%) and partial verification of the reference standard leave residual uncertainty. About three in four patients were classified as “not suspected”, which provides an estimate of the potential reduction in referral workload rather than a directly measured effect on referrals. As referable disease consisted exclusively of moderate non-proliferative DR, these results do not establish performance for severe or proliferative DR. The study findings support the use of point-of-care AI-assisted triage to optimize specialist referrals, but not the use of this algorithm as a stand-alone screening test for DR of any severity. Further prospective, multicentric studies with multi-grader reference standards and adequate representation of severe DR are warranted to confirm these findings and to evaluate the algorithm’s operational and economic impact across broader public health settings.

## Figures and Tables

**Figure 1:**
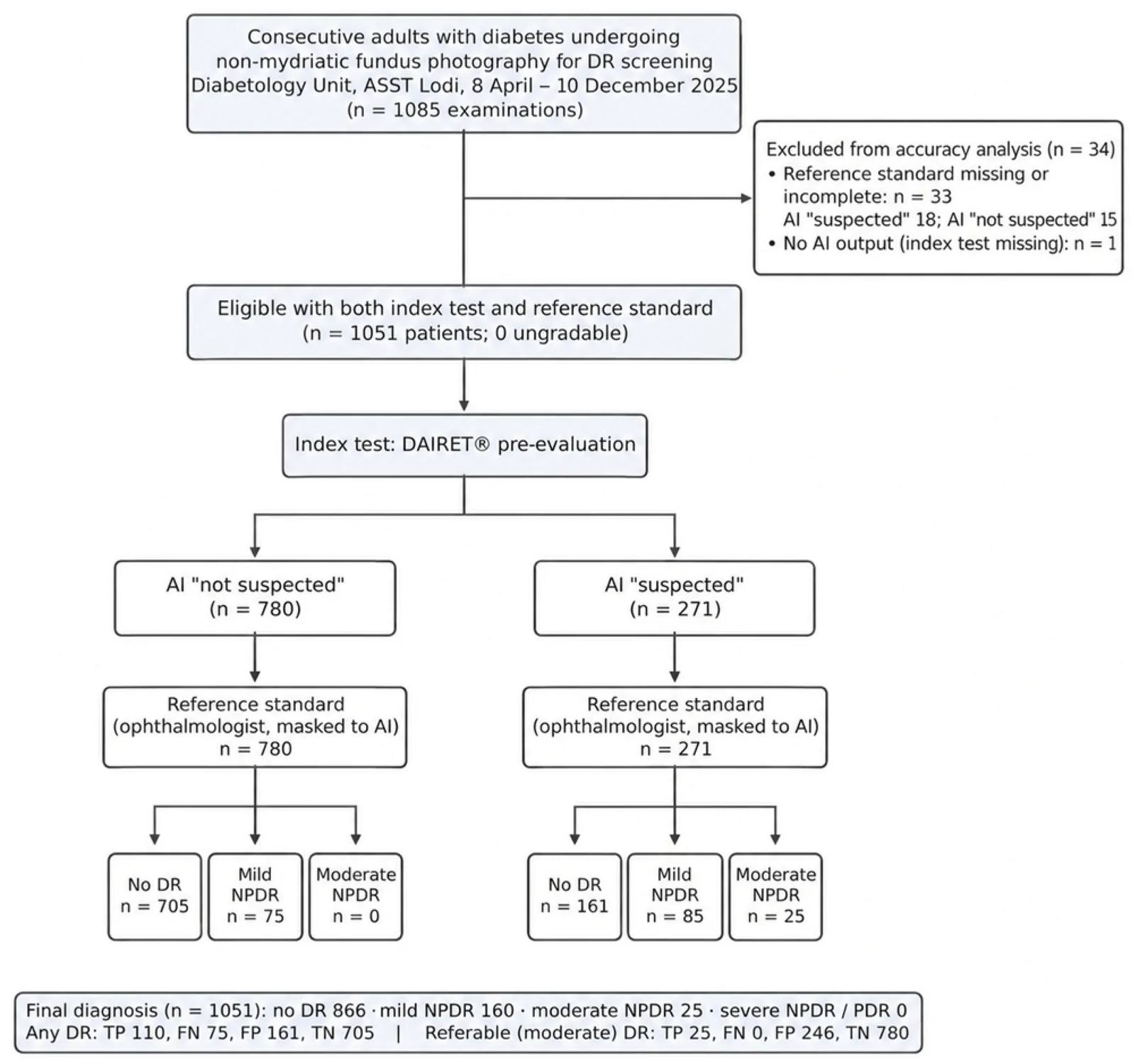
STARD-AI participant flow diagram. Of 1085 consecutive examinations, 34 were excluded because the reference standard was missing or incomplete (n = 33) or the index test output was missing (n = 1), leaving 1051 patients for the accuracy analysis. Patients with a known diagnosis of DR were managed in secondary ophthalmological care and did not enter the screening pathway. DR = diabetic retinopathy; NPDR = non-proliferative DR; PDR = proliferative DR; TP = true positive; FN = false negative; FP = false positive; TN = True Negative.

**Figure 4:**
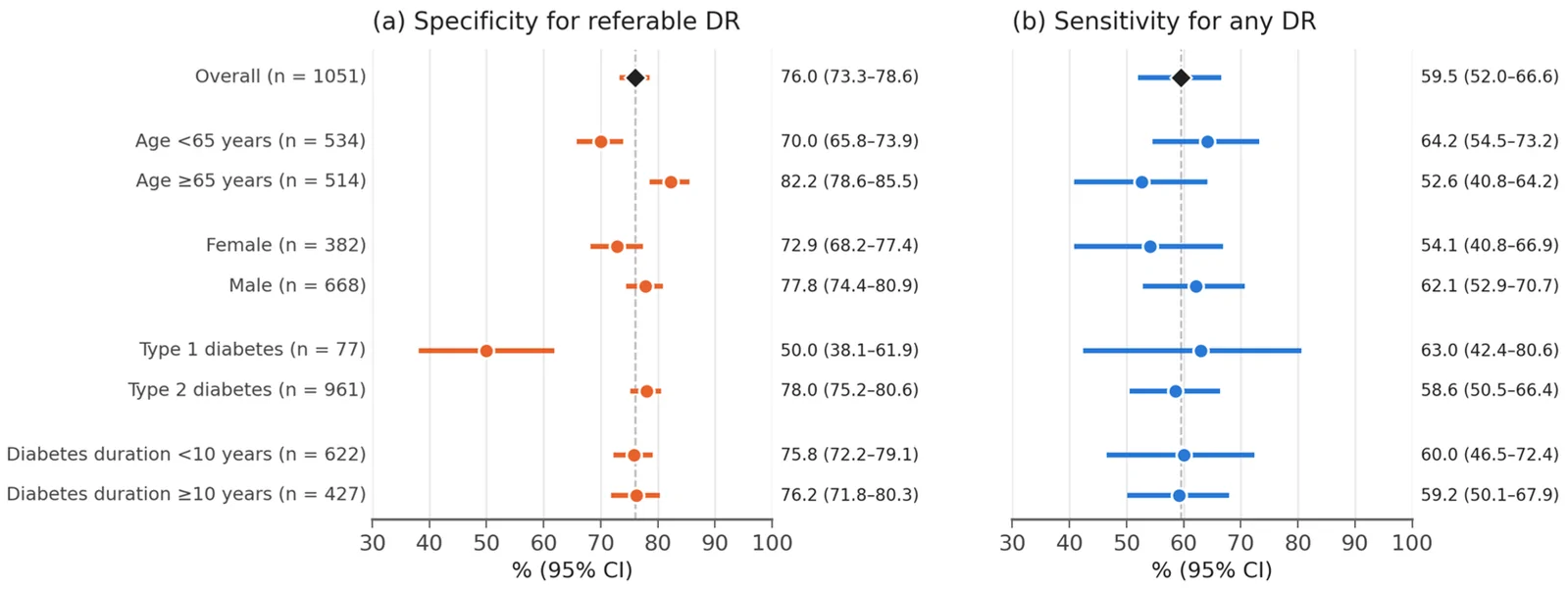
Forest plot of (**a**) specificity for referable DR and (**b**) sensitivity for any DR by clinical subgroup, with exact 95% CIs. The dashed line indicates the overall estimate. Sensitivity for referable DR (100% in all strata) is reported in Table 4.

**Table 1:** Demographic characteristics of the cohort (n = 1051).

Characteristic	Value
Sex, n (%) (n = 1050)	
Male	668 (63.6)
Female	382 (36.4)
Age, years (n = 1048)	
Median (IQR)	64.6 (56.3–72.8)
Mean ± SD	63.8 ± 13.3
≥65 years, n (%)	514 (49.0)
Type of diabetes, n (%)	
T2D	961 (91.4)
T1D	77 (7.3)
Other specific types ^a^	13 (1.2)
Disease duration, years (n = 1049)	
Median (IQR)	7 (2–14)
≥10 years, n (%)	427 (40.7)

T2D = type 2 diabetes; T1D = type 1 diabetes; IQR = interquartile range; SD = standard deviation. ^a^ Pancreatogenic (n = 6) and steroid-induced (n = 7) diabetes. Denominators are reported where covariates were missing (implausible birth dates, n = 3; missing sex, n = 1; non-numeric duration, n = 2).

**Table 2:** Cross-tabulation of AI output (DAIRET^®^) by ophthalmological grade (reference standard).

Ophthalmological Assessment	AI: “Not Suspected” n (Row %)	AI: “Suspected” n (Row %)	Total n (Column %)
Absent DR	705 (81.4%)	161 (18.6%) ^a^	866 (82.4%)
Mild NPDR	75 (46.9%) ^b^	85 (53.1%)	160 (15.2%)
Moderate NPDR (referable)	0 (0.0%)	25 (100%)	25 (2.4%)
Severe NPDR/PDR	0	0	0 (0.0%)
Total	780 (74.2%)	271 (25.8%)	1051 (100%)

DR = diabetic retinopathy; NPDR = non-proliferative DR; PDR = proliferative DR. ^a^ False positives for any DR. ^b^ False negatives for any DR, all restricted to mild NPDR. For referable DR, false positives are the 246 AI-suspected patients without moderate NPDR (161 absent + 85 mild) and false negatives are 0.

**Table 3:** Diagnostic accuracy metrics of the AI algorithm with 95% confidence intervals (n = 1051).

Metric	Any DR % (95% CI); n/N	Referable DR % (95% CI); n/N
Prevalence	17.6% (15.3–20.0) 185/1051	2.4% (1.5–3.5) 25/1051
Sensitivity	59.5% (52.0–66.6) 110/185	100.0% (86.3–100.0) 25/25
Specificity	81.4% (78.7–83.9) 705/866	76.0% (73.3–78.6) 780/1026
NPV	90.4% (88.1–92.4) 705/780	100.0% (99.5–100.0) 780/780
PPV	40.6% (34.7–46.7) 110/271	9.2% (6.1–13.3) 25/271
Overall accuracy	77.5% (74.9–80.0) 815/1051	76.6% (73.9–79.1) 805/1051
Cohen’s κ (95% CI)	0.35 (0.27–0.42)	0.13 (0.04–0.23)
AI “not suspected”	74.2% (71.5–76.8) 780/1051	Same as any DR

DR = diabetic retinopathy; NPV = negative predictive value; PPV = positive predictive value; CI = confidence interval (exact Clopper–Pearson for proportions; asymptotic for κ). Referable DR = moderate or worse DR (in this cohort, moderate NPDR only).

**Table 4:** Diagnostic performance of DAIRET^®^ by clinical subgroup. Values are % (95% CI).

Subgroup	n	Referable Cases, n	AI “Suspected”	Sensitivity, Any DR	Specificity, Any DR	Specificity, Referable DR	Sensitivity, Referable DR
Overall	1051	25	25.8 (23.2–28.5)	59.5 (52.0–66.6)	81.4 (78.7–83.9)	76.0 (73.3–78.6)	100.0 (86.3–100.0)
Age < 65 years	534	18	32.4 (28.4–36.5)	64.2 (54.5–73.2)	75.8 (71.4–79.8)	70.0 (65.8–73.9)	100.0 (81.5–100.0)
Age ≥ 65 years	514	7	18.9 (15.6–22.5)	52.6 (40.8–64.2)	87.0 (83.5–90.0)	82.2 (78.6–85.5)	100.0 (59.0–100.0)
Female	382	5	28.0 (23.6–32.8)	54.1 (40.8–66.9)	76.9 (71.9–81.4)	72.9 (68.2–77.4)	100.0 (47.8–100.0)
Male	668	20	24.6 (21.3–28.0)	62.1 (52.9–70.7)	84.0 (80.7–87.0)	77.8 (74.4–80.9)	100.0 (83.2–100.0)
T1D	77	3	51.9 (40.3–63.5)	63.0 (42.4–80.6)	54.0 (39.3–68.2)	50.0 (38.1–61.9)	100.0 (29.2–100.0)
T2D	961	22	23.8 (21.2–26.7)	58.6 (50.5–66.4)	83.0 (80.2–85.5)	78.0 (75.2–80.6)	100.0 (84.6–100.0)
Other types	13	0	15.4 (1.9–45.4)	100.0 (2.5–100.0)	91.7 (61.5–99.8)	84.6 (54.6–98.1)	NA
Duration < 10 years	622	6	24.9 (21.6–28.5)	60.0 (46.5–72.4)	78.8 (75.2–82.1)	75.8 (72.2–79.1)	100.0 (54.1–100.0)
Duration ≥ 10 years	427	19	27.2 (23.0–31.7)	59.2 (50.1–67.9)	86.1 (81.7–89.8)	76.2 (71.8–80.3)	100.0 (82.4–100.0)

Between group comparison (Fisher’s exact test) for specificity for referable DR: age < 65 years vs. ≥65 years, *p* < 0.001; female vs. males, *p* = 0.082); T1D vs. T2D, *p* < 0.001; duration < 10 years vs. ≥10 years, *p* = 0.940. For specificity for any DR: age, *p* < 0.001; diabetes type, *p* < 0.001; duration, *p* = 0.010. Comparison were not pre-specified and are exploratory. T1D = type 1 diabetes; T2D = type 2 diabetes; other types: pancreatogenic (n = 6) and steroid-induced (n = 7) diabetes. NA = not estimable (no referable cases). NPV for referable DR was 100% in every subgroup. Age was missing for 3 patients, sex for 1 and diabetes duration for 2; these patients were excluded only from the corresponding subgroup.

## Data Availability

The de-identified data presented in this study are available from the corresponding author upon reasonable request. The study protocol is also available from the corresponding author upon request.

## Supplementary Materials

The following supporting information can be downloaded at https://www.mdpi.com/article/10.3390/diagnostics16203422/s1, Table S1. STARD-AI checklist [17]. Item content is paraphrased; see the original publication for the full wording. Table S2. Sensitivity analysis for partial verification: referable diabetic retinopathy (moderate or worse). Values are % (95% CI, exact Clopper–Pearson). Table S3. Sensitivity analysis for partial verification across all 33 patients without a complete reference standard: referable diabetic retinopathy (moderate or worse). Values are % (95% CI, exact Clopper–Pearson).

## Abbreviations

The following abbreviations are used in this manuscript:

AI	Artificial Intelligence
CI	Confidence Interval
CFP	Conventional Fundus Photography
DAIRET	Diabetes Artificial Intelligence for RETinopathy
DFE	Direct Fundus Examination
DM	Diabetes Mellitus
DME	Diabetic Macular Edema
DR	Diabetic Retinopathy
EMR	Electronic Medical Record
F	Female
IQR	Interquartile Range
M	Male
NPV	Negative Predictive Value
NPDR	Non Proliferative Diabetic Retinopathy
OR	Odds Ratio
PPV	Positive Predictive Value
PDR	Proliferative Diabetic Retinopathy
ROC	Receiver Operating Characteristic
SD	Standard Deviation
STARD-AI	Standards for Reporting of Diagnostic Accuracy Studies—Artificial Intelligence
T1D	Type 1 Diabetes
T2D	Type 2 Diabetes
