# Supplementary material for: AI-Assisted Triage for Diabetic Retinopathy Integrated into Routine Diabetes Care: A Real-World Diagnostic Accuracy Study of the DAIRET® Algorithm
Source: Diagnostics (Basel). 2026 Oct 21;16(20):3422. doi: 10.3390/diagnostics16203422 (PMC13703658; doi:10.3390/diagnostics16203422)
Supplement: Supplementary file 1 [file diagnostics-16-03422-s001.zip › diagnostics-4626023-supplementary.pdf]

Table S1. STARD-AI checklist [17]. Item content is paraphrased; see the original publication for the full wording.

Section	Item	Checklist item (paraphrased)	Where reported	Status
Title/Abstract	1	Identification as a diagnostic accuracy study of an AI index test, with at least one accuracy measure	Title; Abstract	Reported
Abstract	2	Structured summary of design, methods, results, conclusions	Abstract	Reported
Introduction	3	Scientific and clinical background, including intended use and clinical role of the AI index test	Introduction; 2.3	Reported
Introduction	4	Study objectives and hypotheses	Introduction (last paragraph)	Reported
Methods – design	5	Prospective or retrospective data collection	2.1	Reported (retrospective)
Methods – ethics	6	Ethics approval or justification	Institutional Review Board Statement	Reported
Methods – participants	7	Eligibility criteria at participant and dataset level	2.1	Reported
Methods – participants	8	Basis on which potentially eligible participants were identified	2.1	Reported (routine screening visits)
Methods – participants	9	Setting, location and dates	2.1; 3	Reported (8 Apr–10 Dec 2025)
Methods – participants	10	Consecutive, random or convenience series	2.1	Reported (consecutive)
Methods – dataset	11	Data source and how data were collected (routine vs study-specific)	2.2	Reported (routine EMR data)
Methods – dataset	12	Who annotated the data (reference standard), experience, context	2.4	Reported
Methods – dataset	13	Acquisition devices, manufacturers, software versions	2.2; 2.3	Reported
Methods – dataset	14	Acquisition protocol and preprocessing	2.2; 2.3	Reported (preprocessing proprietary, declared)
Methods – test methods	15a	Index test in sufficient detail for replication	2.3	Reported (vendor, version, CE class, output, fixed configuration)
Methods – test methods	15b	Development of the index test: training, validation, testing, external evaluation	2.3	Reported (training data from published source); non-disclosed items declared
Methods – test methods	15c	Definition and rationale of positivity threshold; prespecified vs exploratory	2.3	Reported (fixed manufacturer threshold, binary output)
Methods – test methods	15d	Intended end-user and required expertise	2.3	Reported
Methods – test	16a	Reference standard in sufficient detail	2.4	Reported (ICDR scale, per patient,

methods				worse eye)
Methods – test methods	16b	Rationale for choosing the reference standard	2.4; Discussion (limitations)	Reported
Methods – test methods	16c	Definition of reference-standard categories; prespecified vs exploratory	2.4	Reported (referable = moderate or worse, a priori)
Methods – test methods	17a	Whether index-test assessors had access to clinical/reference information	2.3	Reported (image-only AI)
Methods – test methods	17b	Whether reference-standard assessors had access to index-test results	2.4	Reported (masked)
Methods – analysis	18	Methods for estimating diagnostic accuracy	2.5	Reported
Methods – analysis	19	Handling of indeterminate results	2.5; 3.1	Reported (none occurred)
Methods – analysis	20	Handling of missing index-test or reference-standard data	2.1; 2.4; 2.5; 3.6; Table S2	Reported (33 without complete report and 1 without AI output excluded; sensitivity analysis)
Methods – analysis	21	Analyses of variability; prespecified vs exploratory	2.5; 3.4	Reported (exploratory subgroups)
Methods – analysis	22	Intended sample size and how it was determined	2.1	Reported (no formal calculation; precision via CIs)
Methods – analysis	23	Error analysis, algorithmic bias and fairness assessment	2.5; 3.4; 3.5	Reported (FP causes; age/sex/type/duration strata)
Results – participants	24	Participant flow diagram	Figure 1	Reported
Results – participants	25	Baseline demographic, clinical and technical characteristics	Table 1	Reported
Results – participants	26a	Distribution of severity in those with the target condition	3.1; Table 2	Reported
Results – participants	26b	Distribution of alternative diagnoses in those without the target condition	3.5; Table 5	Reported
Results – participants	27	Time interval and interventions between index test and reference standard	2.4	Reported (same images; grading interval reported)
Results – dataset	28	Whether the dataset reflects the expected spectrum in the intended population	3.1; Discussion (limitations)	Reported (no severe DR; upper limit 0.35%)
Results – dataset	29	For external evaluation, differences from development data	2.3; Discussion	Reported (development data partly undisclosed, declared)
Results – test results	30	Cross-tabulation of index test by reference standard	Table 2; Figure 1	Reported
Results – test results	31	Accuracy estimates with 95% CIs	Table 3; Table 4; Figures 3–4	Reported
Results – test results	32	Adverse events from index test or reference standard	—	Not applicable (non-invasive

				imaging)
Discussion	33	Limitations, sources of bias, statistical uncertainty, generalisability	Discussion (limitations)	Reported
Discussion	34	Implications for practice, intended use and clinical role	Discussion; Conclusions	Reported
Discussion	35	Ethical considerations and fairness	Discussion (subgroup paragraph); IRB statement	Reported
Other	36	Registration number and registry	IRB statement	Reported (CET 129-2026; no trial registry)
Other	37	Where the full protocol can be accessed	Data Availability Statement	Reported (on request)
Other	38	Sources of funding and role of funders	Funding	Reported
Other	39	Commercial interests	Conflicts of Interest	Reported
Other	40a	Availability of data and code, restrictions	Data Availability Statement	Reported (data on request; algorithm proprietary)
Other	40b	Whether AI outputs are stored and auditable	2.2; IRB statement	Reported (outputs stored in EMR)

Table S2. Sensitivity analysis for partial verification: referable diabetic retinopathy (moderate or worse). Values are % (95% CI, exact Clopper–Pearson).

**Scenario**	**Sensitivity**	**Specificity**	**PPV**	**NPV**
Primary analysis (n = 1051)	100.0 (86.3–100.0); 25/25	76.0 (73.3–78.6); 780/1026	9.2 (6.1–13.3); 25/271	100.0 (99.5–100.0); 780/780
Partial verification: 1 of the 15 AI-negative patients without a complete reference standard assumed to have referable DR	96.2 (80.4–99.9); 25/26	76.0 (73.3–78.6); 780/1026	9.2 (6.1–13.3); 25/271	99.9 (99.3–100.0); 780/781
Partial verification: 2 of the 15 AI-negative patients without a complete reference standard assumed to have referable DR	92.6 (75.7–99.1); 25/27	76.0 (73.3–78.6); 780/1026	9.2 (6.1–13.3); 25/271	99.7 (99.1–100.0); 780/782
Partial verification: 3 of the 15 AI-negative patients without a complete reference standard assumed to have referable DR	89.3 (71.8–97.7); 25/28	76.0 (73.3–78.6); 780/1026	9.2 (6.1–13.3); 25/271	99.6 (98.9–99.9); 780/783
Partial verification: 15 of the 15 AI-negative patients without a complete reference standard assumed to have referable DR (worst case)	62.5 (45.8–77.3); 25/40	76.0 (73.3–78.6); 780/1026	9.2 (6.1–13.3); 25/271	98.1 (96.9–98.9); 780/795

Specificity and PPV are unchanged across scenarios, because the hypothetical cases are AI-negative patients with referable DR (they enter only the denominators of sensitivity and NPV). The 33 patients without a complete reference standard included 18 AI-positive (54.5%) and 15 AI-negative results. No severe non-proliferative or proliferative DR was observed (0/1051; upper limit of the exact 95% CI 0.35%).

Table S3. Sensitivity analysis for partial verification across all 33 patients without a complete reference standard: referable diabetic retinopathy (moderate or worse). Values are % (95% CI, exact Clopper–Pearson).

Scenario	Sensitivity	Specificity	PPV	NPV
Primary analysis (n = 1051)	100.0 (86.3–100.0); 25/25	76.0 (73.3–78.6); 780/1026	9.2 (6.1–13.3); 25/271	100.0 (99.5–100.0); 780/780
All 33 patients without a complete reference standard assumed to be non-referable	100.0 (86.3–100.0); 25/25	75.1 (72.4–77.7); 795/1059	8.7 (5.7–12.5); 25/289	100.0 (99.5–100.0); 795/795
Reference standard imputed at the prevalence observed within each AI output category (2 of the 18 AI-positive patients referable)	100.0 (87.2–100.0); 27/27	75.2 (72.5–77.8); 795/1057	9.3 (6.2–13.3); 27/289	100.0 (99.5–100.0); 795/795
Best case: all 18 AI-positive patients referable and all 15 AI-negative patients non-referable	100.0 (91.8–100.0); 43/43	76.4 (73.7–78.9); 795/1041	14.9 (11.0–19.5); 43/289	100.0 (99.5–100.0); 795/795
Worst case: all 18 AI-positive patients non-referable and all 15 AI-negative patients referable	62.5 (45.8–77.3); 25/40	74.7 (72.0–77.3); 780/1044	8.7 (5.7–12.5); 25/289	98.1 (96.9–98.9); 780/795

Table S2 varies only the 15 AI-negative patients and therefore leaves specificity and PPV unchanged. Table S3 varies all 33 patients, including the 18 AI-positive ones, and therefore affects all four estimates. In the imputation scenario the reference standard was assigned at the prevalence observed within each AI output category (9.2% among AI-positive and 0% among AI-negative patients), rounded to the nearest patient.
